# When protection becomes a paradox: maternal antibodies clear oral rotavirus vaccines before the infant immune system can learn

**DOI:** 10.1038/s44318-025-00583-1

**Published:** 2025-10-14

**Authors:** Ashomathi Mollin, Stephanie N Langel

**Affiliations:** Department of Pathology, Center for Global Health and Diseases, Case Western Reserve University School of Medicine, Cleveland, OH USA

**Keywords:** Immunology, Microbiology, Virology & Host Pathogen Interaction

## Abstract

New research in *The EMBO Journal* shows that maternal antibodies interfere with oral rotavirus vaccination by accelerating viral clearance.

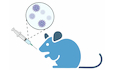

## Teaser

Child morbidity and mortality from infectious diseases remain high in many low- and middle-income countries, highlighting the need for the development of more effective vaccination strategies. In this issue of *The EMBO Journal*, Chandler et al, demonstrate in a neonatal mouse model that maternal antibodies interfere with oral rotavirus vaccination by accelerating viral clearance, offering a mechanistic rationale for reduced vaccine performance in high-burden settings.

Rotavirus (RV) is a leading cause of acute gastroenteritis in children under five. Despite immunization efforts, this pathogen continues to take nearly 200,000 lives annually, especially in low- and middle-income countries, where a combination of host, environmental, and pathogenic factors likely influence the enhanced RV susceptibility and vaccine underperformance (Clark et al, 2017). Infants are born with naïve immune systems, making them vulnerable to infection. Maternal antibodies (MatAbs), including immunoglobulin G (IgG) and immunoglobulin A (IgA), can combat this vulnerability by providing an infant with transient immunity against dangerous pathogens. Maternal IgG is predominantly delivered transplacentally into an infant’s circulation, while IgA is the major antibody passively transferred through breast milk, providing protection at the mucosal surfaces. Through mechanisms like pathogen neutralization, antibody-mediated phagocytosis, antibody-dependent cell-mediated cytotoxicity, and systemic complement activation, these MatAbs can boost the chances of neonatal survival (Langel et al, 2022).

## The paradox of passive protection

Although MatAbs provide early protection, high titers of RV-specific MatAbs from repeated exposures can attenuate vaccine-induced humoral responses in neonates, leaving infants particularly vulnerable during the critical post-weaning period (Chilengi et al, 2016). This phenomenon, termed MatAb interference, has been attributed to multiple, non-mutually exclusive mechanisms, including epitope masking, FcγRIIB-mediated inhibition of B cells, viral neutralization, and antibody-mediated phagocytosis (Niewiesk, 2014). However, no single pathway has emerged as universally responsible, as the extent of interference depends on both the pathogen and vaccine design. In this issue of *The EMBO Journal*, Chandler et al provide compelling evidence from a neonatal mouse model that MatAbs suppress oral rotavirus vaccine immunogenicity by accelerating vaccine clearance, thereby undermining protection (Chandler et al, 2025).

## A novel mouse model of MatAb interference

**Figure 1:**
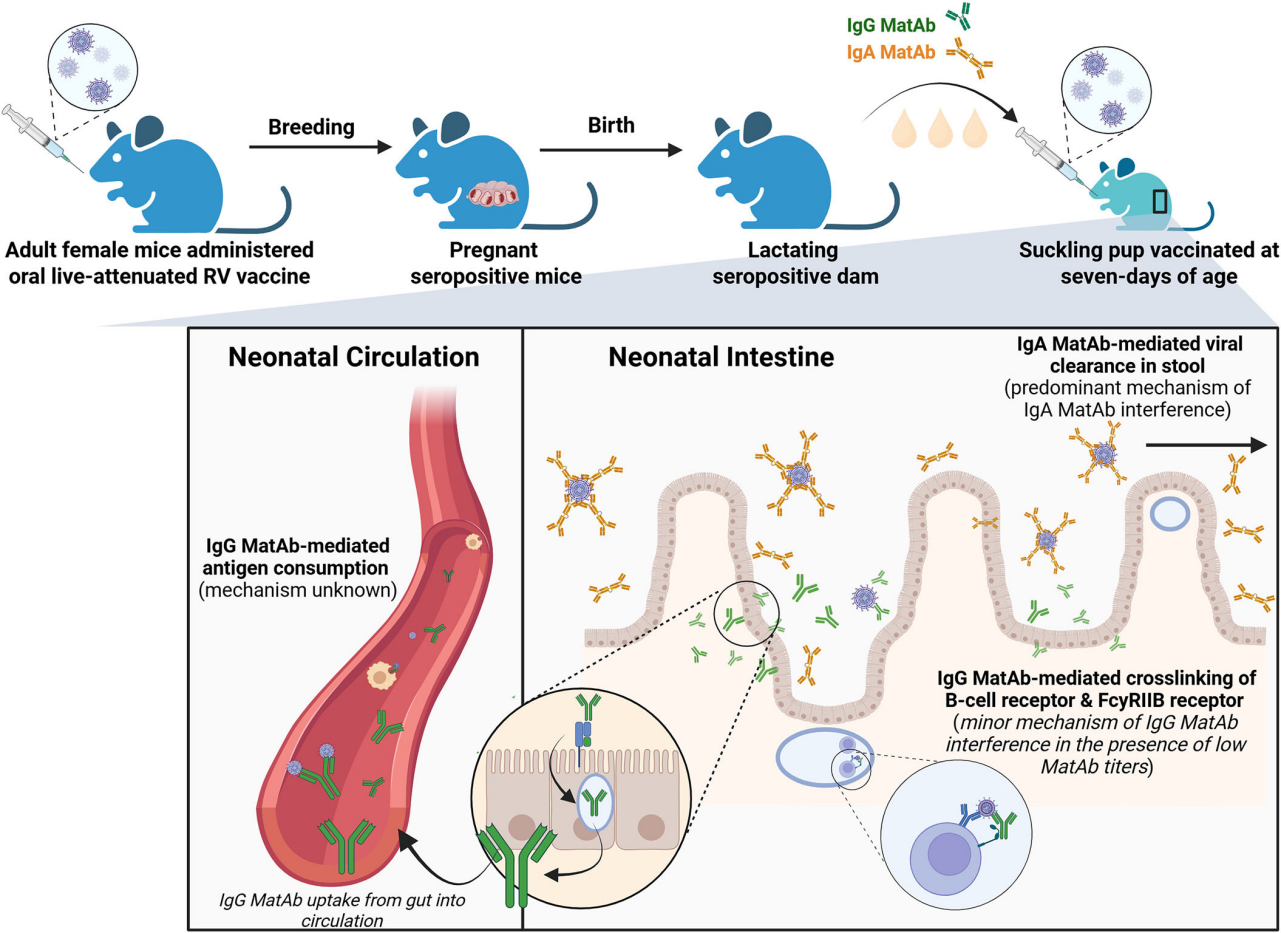
Mechanism of maternal antibody interference. High titers of maternal antibodies (MatAbs) can limit the antigen availability required to generate an infant’s humoral immune response. In this issue, Chandler et al develop an early-life rotavirus (RV) immunization mouse model to elucidate a key mechanism underlying MatAb interference. Maternal RV-specific IgA present in the pup mucosal tract likely blocks viral replication by neutralizing live-attenuated virus particles, as evidenced by reduced viral copy numbers and elevated maternal IgA levels detected in pup stool samples. Similarly, they observed reduced titers of systemic maternal RV-specific IgG in pups, suggesting IgG MatAb-mediated antigen consumption and clearance. In addition, Chandler et al report that FcγRIIB has only a minor influence on pup seroconversion to oral immunization in the presence of low MatAb titers. Figure created with BioRender.

Currently, live-attenuated oral RV vaccines are recommended starting at 6 weeks of age. Adapting this schedule to their mouse model, Chandler et al immunized 7-day-old pups, born to either seropositive or seronegative dams, with a subclinical dose of the homologous live-attenuated murine RV strain, EMcN (Fig. 1). In this setting, pups vaccinated in the presence of RV-specific MatAbs failed to seroconvert, whereas those without MatAbs mounted robust IgG and IgA responses.

A key strength of the study is the ability to distinguish maternal from neonatal antibody responses, which is a challenge in human studies. By crossing C57BL/6 dams with BALB/c males, the authors exploited allelic differences in IgG1 to track whether antibodies in pup serum were maternally derived or produced de novo. The authors found that only pups vaccinated in the absence of MatAbs generated their own IgG1 response. To confirm this, the team analyzed IgA, which is not transferred from milk into pup circulation and therefore any IgA detected in serum or stool must be derived from the pup. Consistently, IgA was detected only in pups without MatAbs, while those exposed to MatAbs showed no IgA induction following vaccination. Importantly, interference persisted even when MatAbs and the vaccine strain were heterologous, underscoring the breadth of the effect.

To test whether lowering MatAb exposure could enhance pup responses, Chandler et al modulated maternal infection dose and passively transferred defined amounts of immune sera to dams. Even very low levels of MatAbs delivered solely through milk were sufficient to block seroconversion in their mouse model.

## Not just fewer antibodies, but fewer germinal centers

What accounts for the absence of antibody production in the presence of MatAbs? The author’s analysis of mesenteric lymph nodes (MLNs) by flow cytometry, histology, and immunofluorescence revealed markedly reduced germinal center (GC) formation in MatAb-exposed pups. Interestingly, T-follicular helper cell frequencies were not significantly altered, indicating that the defect lies primarily within the B-cell compartment. These findings suggest that insufficient antigen availability, rather than a lack of T-cell help, limits the initiation of GC responses and downstream antibody production.

## Minimal role for FcγRIIB signaling

Previous work on MatAb interference implicated inhibitory signaling through FcγRIIB, activated when MatAbs co-ligate FcγRIIB and the B-cell receptor (BCR) (Niewiesk, 2014). In contrast, Chandler et al show that MatAb interference persists in FcγRIIB knockout mice orally vaccinated with high-dose RV. However, at very low MatAb doses, FcγRIIB appears to modestly dampen IgG responses, yet pup IgA production remains consistently suppressed. This novel finding shows that MatAbs differentially regulate IgG and IgA, adding a critical layer of complexity to our understanding of how they shape neonatal, isotype-specific vaccine responses.

## MatAbs mediate vaccine clearance

One of the leading explanations for MatAb interference is direct neutralization of vaccine particles before the neonatal immune system can respond. Chandler and colleagues provide compelling evidence for this mechanism in their mouse model. When pups were vaccinated in the presence of MatAbs, replication of the live-attenuated vaccine in the gastrointestinal tract was significantly reduced. In addition, they demonstrated the enhanced presence of IgA in stool and accelerated waning of maternal IgG in pup serum, a finding that suggests antibody–vaccine complexes cleared during the process. Together, these findings support a model in which both milk-derived IgA and systemically acquired IgG mediate interference through viral clearance. To test whether early clearance had lasting consequences, the investigators challenged mice with RV once MatAbs had waned in adolescence. Expectedly, prior vaccination in the presence of MatAbs conferred no advantage as viral shedding and antibody responses were like those in naïve animals.

Together, these data suggest that high titers of maternally transferred IgG and IgA may limit infant responses to vaccination by rapidly forming immune complexes with vaccine particles and facilitating their clearance.

## Single-cell analysis reveals blunted antiviral programs

To probe how MatAbs reshape neonatal immunity at the cellular level, Chandler et al performed single-cell RNA sequencing of MLN cells from vaccinated pups with or without MatAbs. The overall cellular landscape was similar between groups, but MatAb-exposed pups showed fewer activated and cytotoxic CD8 T cells, consistent with reduced viral replication. Transcriptomic analysis revealed a global downregulation of interferon-α and interferon-γ response pathways across multiple immune subsets, including CD4 T cells, CD8 T cells, plasmacytoid dendritic cells, and B cells.

Pups vaccinated without MatAbs displayed proliferating plasma and germinal center B-cells expressing markers of class-switch recombination and differentiation. In contrast, these populations were sharply reduced in the presence of MatAbs, with diminished expression of key proliferation and activation genes. Instead, naive B-cell signatures were relatively enriched. Together, these data show that MatAbs not only block antibody production but also blunt the broader antiviral gene programs normally induced by vaccination.

## Strategies to overcome MatAb interference

Chandler et al show that even after weaning, circulating IgG MatAbs in the pups continued to block seroconversion, and higher vaccine doses only accelerated antibody consumption without restoring responses. This demonstrates that circulating antibodies alone are sufficient to interfere with immunity in neonatal mice. It also helps explain why withholding breastfeeding in a human study did not substantially affect seroconversion (Rongsen-Chandola et al, 2014), as circulating IgG transferred across the placenta would remain present in neonates to mediate interference. Notably, in the mouse model no vaccine dose was able to overcome MatAb interference when antibody levels were held constant.

The authors next evaluated alternative vaccine designs. A recombinant VP6 subunit vaccine proved ineffective, whereas a double-layered rotavirus particle (DLP) elicited robust IgG responses even in the presence of MatAbs, although it remained less immunogenic than the live-attenuated vaccine. In addition, changing the delivery route proved critical. Parenteral administration of adjuvanted DLPs enabled most pups to overcome MatAb interference and mount protective IgG responses, whereas oral delivery did not. Parenteral subunit approaches are an intriguing alternative for protecting infants where oral vaccines are compromised by MatAbs.

## Limitations and context

All models have inherent limitations. In mice, a smaller fraction of IgG is transferred across the placenta compared to humans, with higher levels delivered through milk (Appleby & Catty, 1983). Therefore, in mice, MatAb interference is primarily mediated by milk-derived antibodies, whereas in humans the relative contribution of transplacental versus breast milk antibodies likely depends on the vaccine platform. Moreover, species-specific differences in rotavirus tropism complicate direct extrapolation across human and murine strains.

While the authors found reducing MatAb titers in pups did not restore neonatal responses, human studies suggest a more graded effect. For example, a mother–infant study in Nicaragua showed that high maternal serum IgG titers correlated with weaker seroconversion, whereas breast milk IgA had no significant impact after the first dose of RV5 (RotaTeq®) (Becker-Dreps et al, 2015). In contrast, another study in Zambia found that elevated breast milk IgA was associated with reduced immunogenicity after the second oral dose of RV1 (Rotarix®) (Chilengi et al, 2016). Moreover, RV vaccine efficacy is consistently lower in cohorts from low- and middle-income countries compared to cohorts from high-income countries, despite similar MatAb titers (Parker et al, 2021). Taken together, these data suggest that in humans, MatAbs are more likely to attenuate rather than completely block seroconversion. In addition, factors common in low- and middle-income countries, such as high multi-pathogen burden and enteric enteropathies, which were not assessed in this study, likely further contribute to reduced vaccine efficacy.

Another important distinction is that Chandler et al were unable to identify a vaccine dose sufficient to overcome MatAb interference in their mouse model. In contrast, infection with the wild-type murine RV strain EDIM has been shown to bypass MatAbs, mounting delayed yet protective humoral immunity after weaning (Muleta et al, 2024). This discrepancy likely reflects differences in viral potency, with the attenuated EMcN vaccine strain used by Chandler et al being less able to overcome MatAb blockade than the more virulent EDIM strain. These findings underscore the relevance of this present study for understanding why attenuated RV vaccines may underperform in endemic settings.

Lastly, additional milk-derived factors, including innate immune components and other immune cells, could also affect MatAb interference, while they were not assessed in this study.

## A broader immunological lesson and outlook

This study demonstrated in a mouse model that MatAb interference with attenuated RV vaccination is not primarily a cell-intrinsic inhibitory process but rather a biophysical one, in which antigen is neutralized too rapidly to initiate effective immune priming. Single-cell analysis highlighted a potential mechanism in which the absence of live-attenuated virus particles limits replication and stimulation of interferon responses, and without that antiviral landscape, germinal centers fail to form.

The mechanistic insight provided by Chandler et al offers a guiding principle for RV vaccines, which is to either prevent vaccine neutralization in the gut lumen or bypass the gut entirely with a parenteral vaccine. As next-generation vaccine platforms advance, incorporating assessments of MatAb interference into preclinical evaluation will be essential for accurately predicting efficacy in infants. Only then can we distinguish vaccine candidates that appear potent in naïve models from those capable of protecting infants born with RV-specific maternal immunity.
